# Bilateral lateral ventricular epidermoid cyst: A case report

**DOI:** 10.1016/j.ijscr.2025.111944

**Published:** 2025-09-15

**Authors:** Bizuayehu Asefa, Eyob Zenebe, Ermias Feleke, Worku Furi, Henock Solomon

**Affiliations:** PO Box: 9086, Addis Ababa University, School of Medicine, Depart of Surgery, Neurosurgery Unit, Ethiopia

**Keywords:** Epidermoid cyst, Inclusion cyst, Lateral ventricle, Case report, Hydrocephalus, Intracranial

## Abstract

**Introduction:**

Epidermoid cysts are benign inclusion cysts that arise from ectopically displaced ectodermal tissue. Intraventricular epidermoid cysts are uncommon, and involvement of the bilateral lateral ventricles is rarely reported. Computed tomography (CT) scans typically show well-localized, hypodense lesions. These cysts are slightly hyperintense to cerebrospinal fluid (CSF) on both T1 and T2 magnetic resonance imaging (MRI) sequences.

**Case presentation:**

We report a 17-year-old male diagnosed with bilateral lateral ventricular epidermoid cysts after presenting with a four-year history of episodic generalized tonic-clonic seizures and recurrent throbbing global headache. He had surgery, and histopathology confirmed an epidermoid cyst. Postoperatively, the patient experienced symptom improvement.

**Discussion:**

Intracranial epidermoid cysts are benign, accounting for 0.2 %–1.8 % of intracranial tumors. Lateral ventricular epidermoids present with obstructive hydrocephalus and signs and symptoms of increased intracranial pressure. Pathological analysis reveals a pearly white tumor composed of simple squamous cells with abundant laminated and compacted keratin and positivity for epithelial membrane antigen. Both microscopic and endoscopic techniques can be used for the resection of lateral ventricular epidermoid cysts.

**Conclusion:**

Lateral ventricular epidermoids are rare benign lesions. Clinical features include symptoms of increased intracranial pressure, such as headache, vomiting, and altered mentation. MRI is the diagnostic imaging modality of choice. Complete surgical resection is curative, with rare reports of recurrence after subtotal resection. Follow-up is crucial to monitor for recurrence and other associated complications.

## Highlights

• Epidermoid cysts are benign inclusion cysts that arise from ectopically displaced ectodermal tissue
• Involvement of the ventricles in epidermoid cyst is uncommon and bilateral involvement is rarely reported.
• The diagnosis of epidermoid cyst is based on typical CT and MRI findings coupled with histopathological confirmation.
• Complete surgical resection is curative with rare reports of recurrence after subtotal resection.
• Long-term follow-up is crucial to monitor for recurrence and associated complications.

## 1 Introduction

Epidermoid cysts are benign inclusion cysts that originate from the ectopic displacement of ectodermal tissue during the disjunction of the neural tube and overlying ectoderm between the 3rd and 5th weeks of embryonic life. Rarely, acquired implantation following trauma or surgical procedures may be the cause [1]. Epidermoid cysts account for 0.2 %–1.8 % of primary intracranial tumors and are more common than other inclusion cysts, namely neurenteric cysts and dermoid cysts [2].

Epidermoid cysts can occur anywhere along the neuraxis, most commonly in the cerebellopontine angle (40 %–50 %), followed by the parasellar region (30 %). Among the ventricles, the fourth ventricle is the most common location, accounting for 5 %–31 % of all epidermoid cysts [3].

The usual age of presentation for epidermoid cysts is between 19 and 69 years. Childhood onset of symptoms is uncommon. Intraventricular cysts typically present with signs and symptoms of intracranial hypertension, obstructive hydrocephalus, and recurrent episodes of aseptic meningitis [4,5].

Radiological evaluation with CT and MRI remains the cornerstone of diagnosis. Brain CT generally shows well-defined, low-density lesions, with calcification observed in 10 %–25 % of cases [2]. The presence of high lipid content, such as triglycerides or cholesterol breakdown products within the cyst, may result in fat attenuation on CT. Although imaging results may vary depending on cyst content, most cases are characteristically slightly hyperintense to CSF on both T1 and T2 MRI sequences. They exhibit high signal intensity on DWI, incomplete suppression on T2-FLAIR, and no post-contrast enhancement [2,6].

In this report, we describe a 17-year-old male diagnosed with bilateral lateral ventricular epidermoid cysts.

This case report has been prepared in accordance with the SCARE guidelines [7].

## 2 Case

A 17-year-old male presented with a history of episodic generalized tonic-clonic seizures and recurrent, throbbing global headache of four years' duration. Three weeks before admission, he developed progressive weakness in all four extremities, along with urinary and fecal incontinence and altered mentation. He was prescribed oral phenobarbital 100 mg daily but discontinued the medication three months prior to presentation.

On neurological examination, his Glasgow Coma score was 13/15, with spontaneous eye opening, production of inappropriate words, and ability to obey commands. Motor examination revealed decreased muscle strength in all four extremities (power 3/5), increased muscle tone, and hyperreflexia of +4 deep tendon reflexes.

**Fig. 1:**
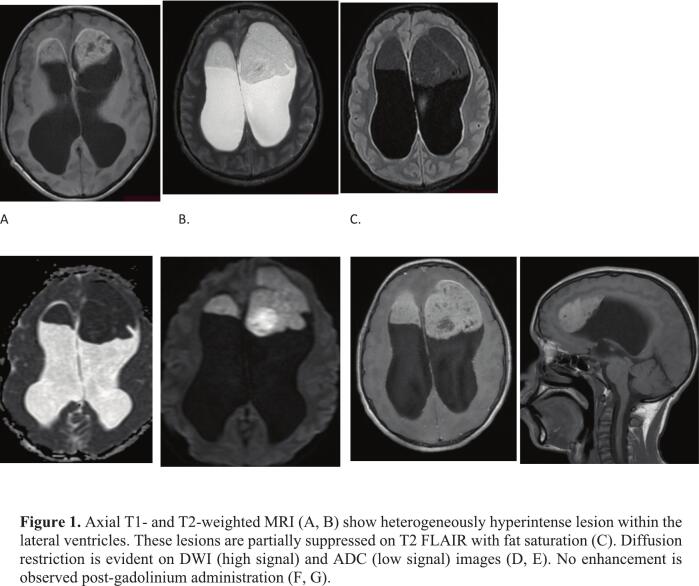
Axial T1- and T2-weighted MRI (A, B) show heterogeneously hyperintense lesion within the lateral ventricles. These lesions are partially suppressed on T2 FLAIR with fat saturation (C). Diffusion restriction is evident on DWI (high signal) and ADC (low signal) images (D, E). No enhancement is observed post‑gadolinium administration (F, G).

**Fig. 2:**
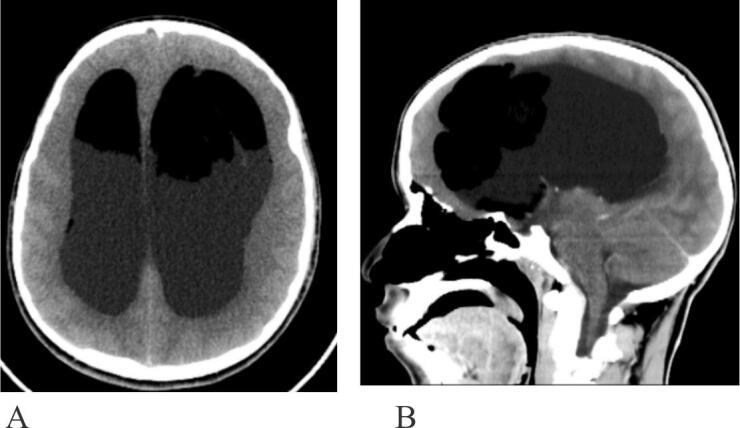
Axial CT without contrast (A) and sagittal CT with contrast (B) show fat attenuation of the lesion and associated hydrocephalus.

A brain MRI, performed one week before surgery, revealed a heterogeneously hyperintense lesion anteriorly within the lateral ventricles on T1 and T2 sequences, with partial suppression on T2-FLAIR imaging performed with fat saturation (Fig. 1A–C). Additionally, diffusion restriction was noted on DWI/ADC images (Fig. 1D, E). The lesions did not show enhancement after gadolinium administration (Fig. 1F, G). A brain CT scan revealed bilateral frontal horn fat-attenuating lesions with hydrocephalus (Fig. 2A, B). The lesion measured 5.7 × 6.4 × 6.3 cm on the left and 4.0 × 3.1 × 4.0 cm on the right side.

The patient underwent a bifrontal craniotomy and transcortical-transventricular approach. An incision was made over the bilateral frontal lobes to access both frontal horns separately. Upon opening the ependymal lining, cerebrospinal fluid (CSF) escaped, and the tumor was seen, confirming its intraventricular location. Microsurgical resection was performed, first resecting the left lateral ventricular tumor, followed by the right side.

**Fig. 3:**
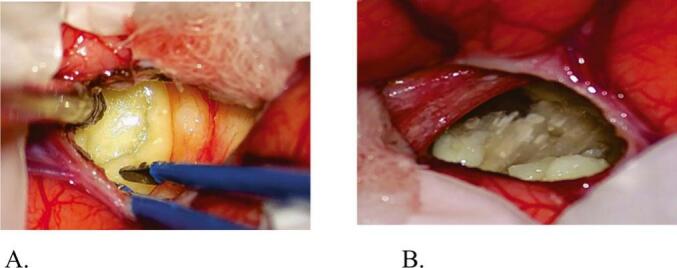
Intraoperative images (A, B) show a whitish, soft-to-firm mass floating in the lateral ventricles with a peripheral capsule adhering to the ependymal lining.

Intraoperatively, an avascular, loosely arranged, flaky, whitish, soft-to-firm mass was observed floating in the ventricles, with a peripheral capsule attached to the ependymal lining of the roof and medial wall of the frontal horns, extending to the foramen of Monro and causing bilateral obstruction (Fig. 3A, B). Thorough internal debulking of the tumor was performed under continuous irrigation of the surgical field with Ringer's lactate mixed with hydrocortisone. Hydrocortisone was used to decrease the risk of aseptic meningitis.

**Fig. 4:**
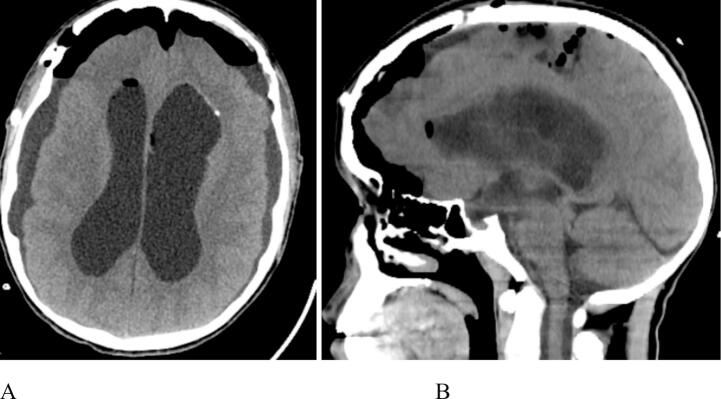
Postoperative axial [A] and sagittal [B] CT scans without contrast show near-total tumor removal with a small remnant attached to the ventricular wall. The scans also reveal a marked reduction in ventricular size, bilateral frontal pneumocephalus, and bilateral subdural collections, which were managed with a temporary subdural drain.

After tumor resection, unobstructed flow of CSF was witnessed through both foramena of Monro. No extension of the mass into the brain parenchyma was noted. After surgery the patient was extubated and transferred to ICU for overnight monitoring. A postoperative CT scan showed near-total tumor resection, with a small remnant attached to the ventricular wall. Additionally, it revealed a marked reduction in ventricular size and bilateral subdural collections (Fig. 4A, B). A temporary subdural drain was inserted and removed on the second postoperative day. Insertion of a permanent ventricular shunt was deferred because the obstruction at the foramen of Monro was addressed intraoperatively.

**Fig. 5:**
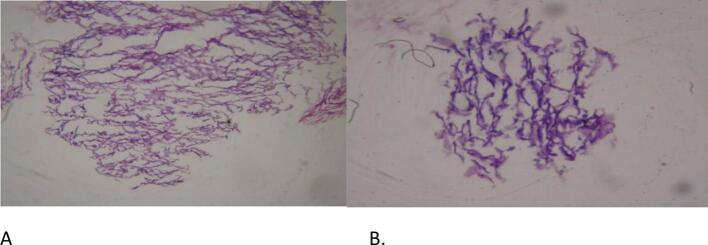
Medium [A] and high [B] power histopathology images show keratin flakes admixed with anucleate squamous cells, consistent with an epidermoid cyst.

Postoperatively, the patient's mental status improved to GCS 15 on the 3rd postoperative day. Sutures were removed after 10 days. The patient was subsequently evaluated in the referral clinic at 1 and 3 months. At the 3-month follow-up, the patient was communicating well, with improved weakness and performance status. Tissue samples were sent for histopathological analysis, which revealed keratin flakes admixed with scanty anucleate squamous cells, confirming a benign pathology consistent with an epidermoid cyst (Fig. 5A, B).

## 3 Discussion

Intracranial epidermoid cysts, also known as pearly tumors, are benign cysts accounting for 0.2 %–1.8 % of intracranial tumors. Intraventricular epidermoid cysts are uncommon, with a lateral ventricular location being extremely rare [5].

Due to their insidious growth pattern and expansion into the lateral ventricle, these tumors typically present in the fifth or sixth decade of life [8,9]. The earlier presentation in our 17-year-old patient could be attributed to bilateral obstruction of the foramen of Monro, causing obstructive hydrocephalus and symptoms of increased intracranial pressure.

As a result of the rarity of lateral ventricular epidermoid cysts, their clinical presentation is not clearly defined. Intraventricular epidermoid cysts typically present with symptoms of increased intracranial pressure, such as altered mentation, vomiting, and headache, resulting from hydrocephalus secondary to obstruction of the foramen of Monro [10]. Our patient presented with throbbing headache, vomiting, abnormal body movements, behavioral changes, and urinary incontinence. These latter symptoms can be attributed to a significant mass effect on both frontal lobes.

The presence of cellular debris and high cholesterol content reduces the density of epidermoid cysts, making it challenging to differentiate them from cerebrospinal fluid (CSF). On MRI, these lesions are characteristically isointense or slightly hyperintense to CSF on both T1- and T2-weighted spin echo images without contrast enhancement [10]. In the ventricles, their signal similarity to CSF on T2-weighted imaging can be distinguished by absent or incomplete suppression on FLAIR images. Epidermoid cysts can be readily differentiated from other cystic masses, such as arachnoid cysts, and distinguished from CSF using diffusion-weighted imaging [6,11].

Epidermoid cysts expand through the accumulation of keratin and cholesterol, which are generated as byproducts from the shedding of epithelial cells. Immunohistochemistry shows positivity for epithelial membrane antigen but no reactivity for glial fibrillary acidic protein or S-100 [5,7].

Complete surgical resection is considered the standard of care for these lesions due to reports of late recurrences requiring repeat surgery [6]. Additionally, there is a report of squamous cell carcinoma following subtotal resection of a lateral ventricular epidermoid cyst [12].

The principles of surgery for lateral ventricular epidermoid cysts align with those for epidermoid cysts in other locations, namely: internal debulking of the soft contents, removal of the cyst lining, protection of the ependyma, avoidance of damage to the subependymal tissue, and prevention of cyst content leakage into the intraventricular space to reduce the risk of chemical ventriculitis and delayed postoperative hydrocephalus. If significant adhesion exists between the cyst lining and the ependymal wall, internal decompression of the cyst can be performed without removing the adherent cyst wall, and this approach has no significant impact on overall survival [8].

Aseptic meningitis is a known complication following epidermoid cyst surgery and can be treated with serial lumbar punctures and corticosteroids [13]. In our patient, a high preoperative suspicion of this complication, combined with thorough removal of the cyst contents under continuous irrigation of the surgical field with Ringer's lactate mixed with hydrocortisone, was performed to prevent aseptic meningitis.

Regular postoperative follow-up is essential to monitor for rare cases of recurrence and other associated complications, such as delayed hydrocephalus.

## 4 Conclusion

Lateral ventricular epidermoids are rare, benign lesions. Clinical features include symptoms of increased intracranial pressure, such as headache, vomiting, and changes in mentation. MRI is the diagnostic imaging modality of choice, typically showing a lesion that is slightly hyperintense relative to CSF on both T1- and T2-weighted sequences, with absent or incomplete suppression on FLAIR images and diffusion restriction on diffusion-weighted imaging sequences. Complete surgical resection is curative, with recurrence rarely reported after subtotal resection. Follow-up is crucial to monitor for recurrence and other associated complications.

## Ethical approval

Ethical approval was provided by the author's institution.

## Funding

None.

## Author contribution

Bizuayehu Asefa Tarekegn, MD, neurosurgery resident: conceived, wrote, and submitted the report. Involved in the diagnosis, management and follow up of the patient.

Eyob Zenebe, MD, Assistant professor of Neurosurgery: Operated on the patient. Reviewed the case report.

Ermias Feleke Alemu, MD, neurosurgery resident: Reviewed the case report and Involved in the diagnosis, management and follow up of the patient

Worku Furi, MD, Neuro-radiology fellow: Involved in examining the CT/MRI and reviewing the case report

Henock Solomon, MD, Pathology resident: Involved in examining the pathology specimen and reviewing case report

## Guarantor

Abdulsemed Mohammed, MD, Assistant professor of Neurosurgery: Reviewed the case report.

Tadelu Mekonnen, MD, Neurosurgery resident: Involved in writing the case report.

## Research registration number

Not Applicable

## Patient (parent's) consent

Written informed consent was obtained from the patient parents for publication of this case report and accompanying images. A copy of the written consent is available for review by the Editor-in-Chief of this journal on request.

## Declaration of Generative AI and AI-assisted technologies in the writing process

During the preparation of this work, the authors used Grok 3, created by xAI, in order to assist with spelling and grammar checks. After using this tool/service, the authors reviewed and edited the content as needed and take full responsibility for the content of the publication.

## Conflict of interest statement

All authors declare that they have no conflict of interest.
